# Incidental apical left ventricular mural thrombus late after myocardial infarction with chronic myeloid leukaemia detected by cardiac magnetic resonance: a case report

**DOI:** 10.1093/ehjcr/ytaf615

**Published:** 2025-12-23

**Authors:** Qui Minh Nguyen, Fajer Almoosa, Mindy Chu Ming Choong, Mamdouh Elsmaan, Francisco Diogo Alpendurada

**Affiliations:** Cardiovascular Magnetic Resonance Unit, Royal Brompton Hospital, Sydney Street, London, SW3 6NP, UK; Cardiovascular Magnetic Resonance Unit, Royal Brompton Hospital, Sydney Street, London, SW3 6NP, UK; Cardiovascular Magnetic Resonance Unit, Royal Brompton Hospital, Sydney Street, London, SW3 6NP, UK; Cardiovascular Magnetic Resonance Unit, Royal Brompton Hospital, Sydney Street, London, SW3 6NP, UK; Cardiovascular Magnetic Resonance Unit, Royal Brompton Hospital, Sydney Street, London, SW3 6NP, UK

**Keywords:** Mural thrombus, Myocardial infarction, Chronic myeloid leukaemia, Cardiovascular magnetic resonance imaging, Case report

## Abstract

**Background:**

Left ventricular (LV) thrombus represents a critical complication following acute myocardial infarction (MI), particularly in patients with reduced LV ejection fraction (LVEF) < 40%, extensive MI, and LV aneurysm. Chronic myeloid leukaemia (CML), a haematological malignancy, is a recognized risk factor for thrombosis. While transthoracic echocardiography (TTE) serves as a ‘goalkeeper’ for the initial screening modality, its sensitivity in detecting mural thrombi remains limited. Cardiac magnetic resonance (CMR) with gadolinium enhancement has emerged as the diagnostic gold standard due to its superior spatial resolution and ability to recognize thrombus.

**Case summary:**

A 47-year-old male with CML, undergoing chemotherapy and a history of extensive anterior ST elevation myocardial infarction (STEMI) treated with primary percutaneous coronary intervention (PCI) of the proximal left anterior descending (LAD) coronary artery, presented with exertional dyspnoea. Although serial non-contrast TTEs over 12 months revealed reduced left ventricular ejection fraction (LVEF) and extensive regional wall motion abnormalities, with pulmonary hypertension (PASP 46 mmHg), no thrombus was detected. Contrast-enhanced CMR identified a large transmural MI involving the anterior, anterolateral walls, and apex with an apical LV mural thrombus. After 3 months of warfarin therapy, the thrombus reduced in size and showed no improvement in LV systolic function (LVEF 36%).

**Conclusion:**

CMR is superior to non-contrast TTE in detecting and monitoring LV thrombus treatment. The early CMR should be considered in high-risk patients post MI, particularly in those with concomitant haematological malignancy.

Learning points

- Chronic myeloid leukaemia, a haematological malignancy, is a recognized risk factor for thrombosis.
- Early CMR should be considered in post-MI patients for screening LV thrombus in high-risk patients, especially those with a coexisting haematological malignancy, regardless of the negative echocardiography.

## Introduction

LV thrombus is a recognized adverse complication following acute MI.^1^ CML, a haematological malignancy characterized by a neoplastic disorder of myeloid cell lines, is a recognized risk factor for thrombus formation.^2^ Although advancements in medical therapy have substantially reduced its incidence in recent decades, the rate remains considerable.^2,3^ While TTE is recommended as a first-line imaging modality for LV thrombus screening, it has inherent limitations.^4^ CMR imaging offers superior sensitivity for thrombus detection. We herein report a case of late LV thrombus identification by CMR in a patient with a history of MI and CML, despite multiple previous TTE examinations that were unrevealing.

## Summary figure

**Figure:**
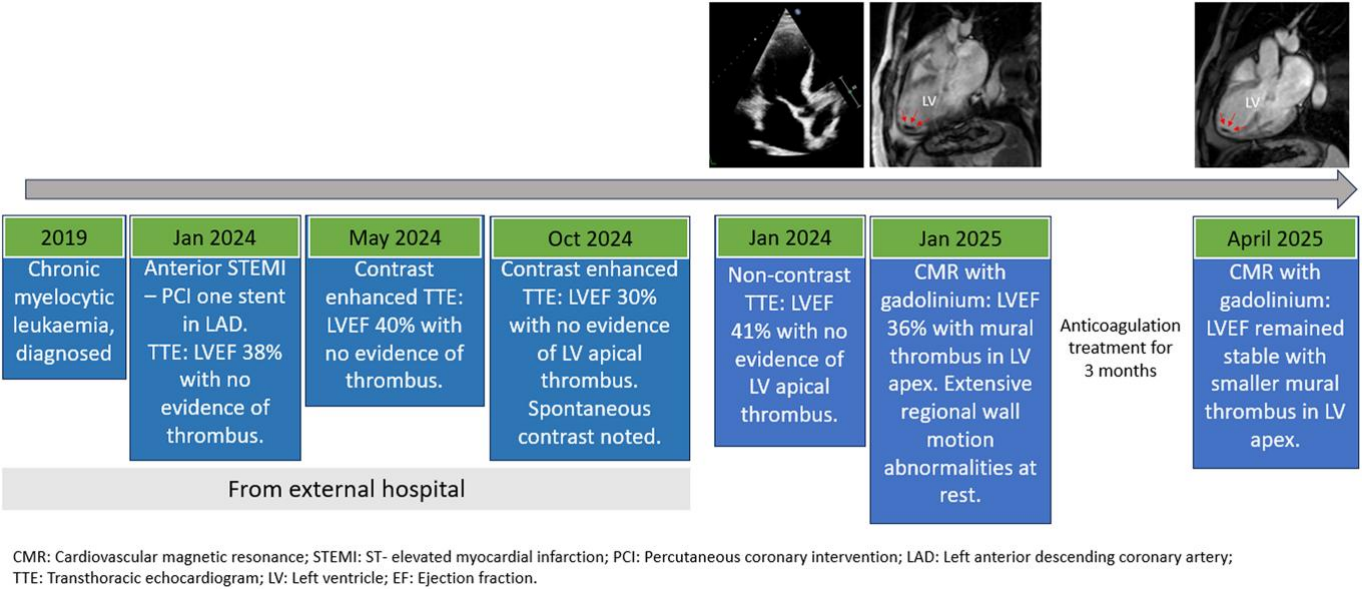


## Case presentation

A 47-year-old male with a history of CML (on chemotherapy and under consideration for stem cell transplantation) and prior STEMI treated with primary PCI in the proximal LAD artery presented with dyspnoea on exertion. Serial TTEs over the past year have been performed for surveillance.

**Figure 1:**
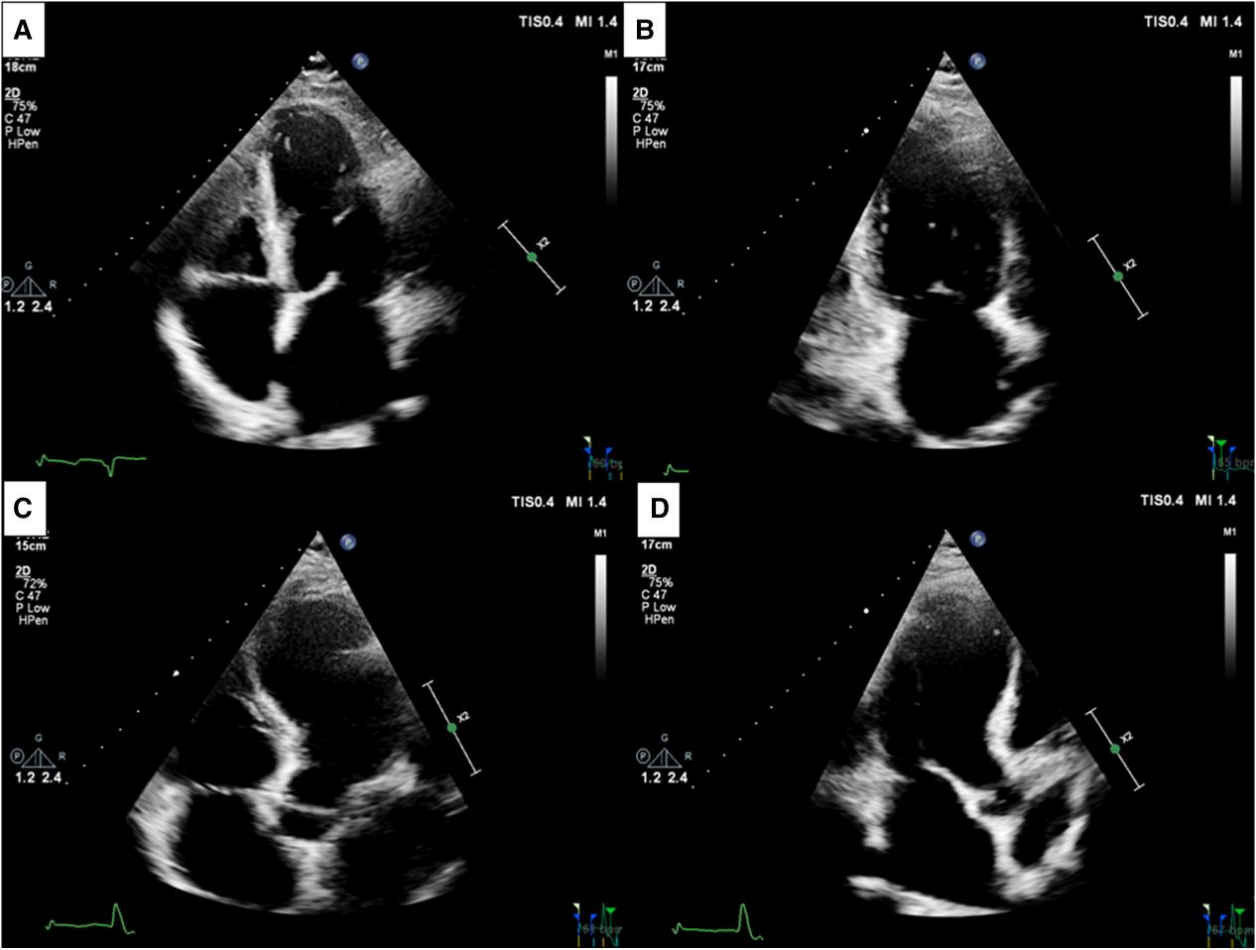
Transthoracic echocardiographic images in the four-chamber (*A*), two-chamber (*B*), five-chamber (*C*), and three-chamber apical views (*D*).

An electrocardiogram revealed sinus bradycardia and a persistent anteroseptal infarct pattern consistent with previous recordings. The recent non-contrast TTE performed to assess cardiac function demonstrated biatrial enlargement, mild-to-moderate mitral regurgitation, elevated pulmonary artery pressure (PAPS 46 mmHg), and severely reduced LVEF with extensive regional wall motion abnormalities. Right ventricular systolic function remained within normal limits. However, the apical echocardiographic window was suboptimal, with no evidence of thrombus (*Figure 1*).

**Figure 2:**
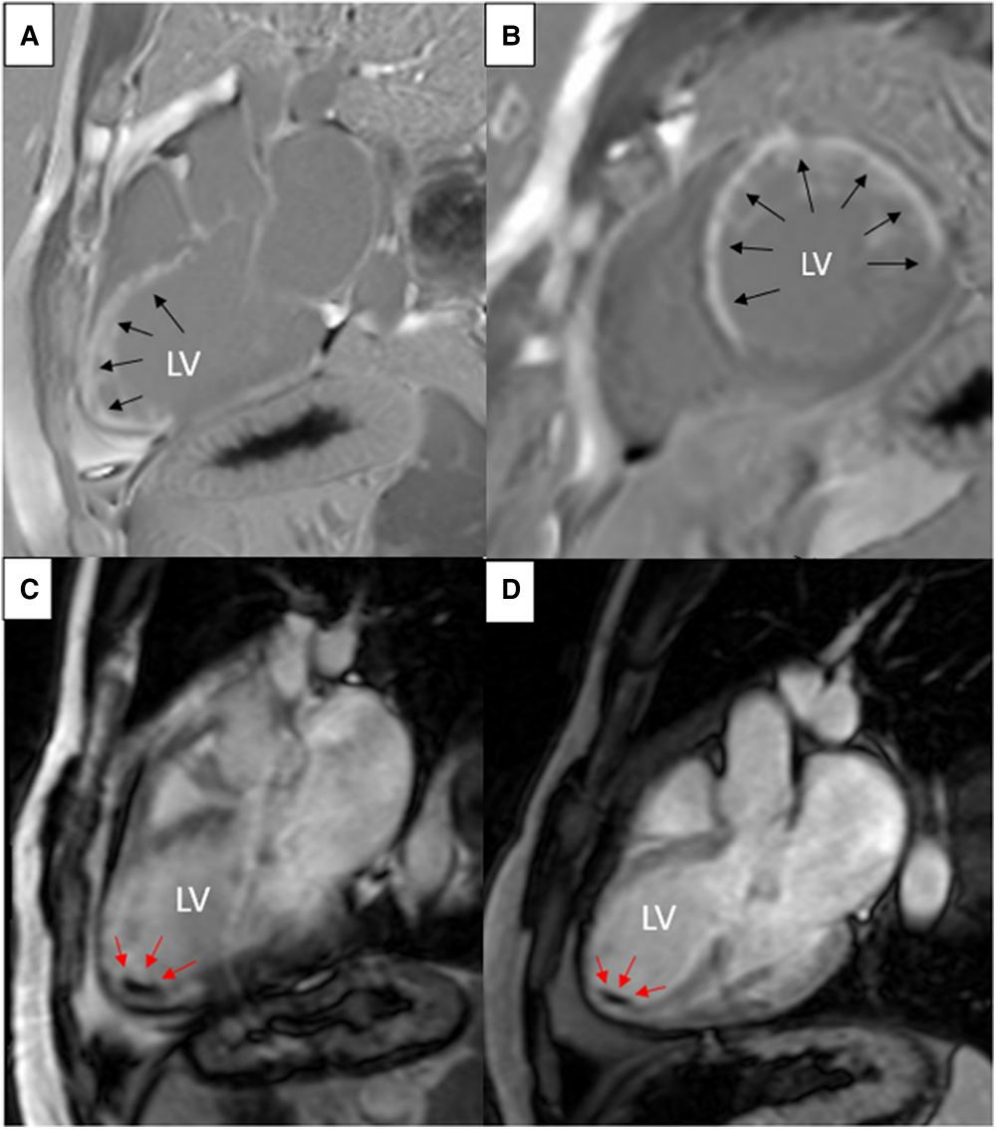
The CMR images show extensive myocardial infarction (*A* and *B*) and thrombus at the apex of the left ventricle before (*C*) and after (*D*) anticoagulant treatment.

Then, CMR was performed to assess cardiac function and myocardial ischemia. Cine-CMR imaging revealed LV dilatation and severely impaired systolic function, with no obvious evidence of thrombus. Early (EGE) and late gadolinium enhancement (LGE) images showed an incidental mural thrombus at the LV apex (*Figure 2C*). Additionally, transmural MI was observed in the basal anteroseptal and anterior walls, mid-ventricular anterior, septal, and lateral walls, and all apical segments (*Figure 2A* and *B*). After three months of treatment with warfarin, contrast-enhanced CMR demonstrated reduced thrombus size (*Figure 2D*) with severely reduced LV ejection fraction (LVEF 36%).

## Discussion

LV thrombus incidence has declined with reperfusion advances: from 17% post-MI (34–57% anterior MI) in the thrombolytic era,^5^ to 2.7%—3.5% overall (7.1%—9.1% anterior STEMI) with primary PCI,^2,3^ reflecting advances in reperfusion and optimized antithrombotic strategies.^4^ CML, a hematologic malignancy characterized by unregulated growth of myelogenous leukocytes.^6^ Although systemic thrombosis is well-documented in CML, intracardiac involvement is rare.^6^

Patients with large or anterior MI (particularly those with delayed reperfusion), LVEF <40%, or LV aneurysm formation constitute the highest-risk population for apical thrombus development.^1,7,8^ These risk factors reflect the interplay of blood stasis (from impaired contractility), endocardial injury, and prothrombotic states as described in Virchow's triad, with the GISSI-3 substudy providing robust epidemiological support for LVEF as an independent predictor.^8,9^ This case presented multiple established risk factors for thrombus formation, including extensive MI (involving the anterior wall) and severely reduced LVEF. Furthermore, the presence of CML may have acted synergistically in this case.^10^

Current guidelines recommend TTE as the primary imaging modality for LV thrombus detection.^4^ However, when suboptimal apical visualization, anterior/apical wall motion abnormalities (WMA), or elevated apical WMA scores (≥5 on non-contrast TTE) are present, advanced imaging with contrast-enhanced TTE or CMR should be considered based on institutional capabilities.^7^ For high-risk patients without initial thrombus identification, contemporary evidence supports surveillance imaging (TTE or CMR) at 1–3 months post-MI.^7^ Therefore, serial non-contrast TTE represents a clinically appropriate strategy for both thrombus surveillance and cardiac function monitoring in this case. Unfortunately, during the 12-month follow-up period, no evidence of LV thrombus was identified in this case on echocardiography, despite multiple thorough assessments. While echocardiography readily identifies large, protuberant, or mobile thrombi, it often fails to detect those with opposing characteristics.^11^ Furthermore, delayed LV thrombus detection after MI on CMR may increase the risk of systemic embolization, as identified by echocardiography.^11^

There is a variety of evidence showing the limitations of TTE in assessing thrombus, particularly the mural subtype. Mural thrombus frequently evades detection on non-contrast TTE due to its structural integration. Contrast-enhanced CMR demonstrates superior thrombus detection capability compared to Cine-CMR and emerges as the diagnostic gold standard for this subtype.^12^ It proves superior sensitivity (90–95%) compared to non-contrast TTE (sensitivity 23–37%), contrast-enhanced TTE (sensitivity 54–64%), and transoesophageal echocardiography (sensitivity 40%).^1,4,12–14^ Characteristic findings of LV thrombus include a hypointense intracardiac mass on cine sequences, adjacent to regional wall thinning and/or akinesis, along with a homogeneous low-signal mass adherent to hyperenhanced scar tissue on EGE and LGE images.^14,15^ These features are compatible with our patient. No embolic complications were noticed during our patient's monitoring, generally occurring in 2.0–6.1% of patients with LV thrombus.^1,14^ Moreover, anticoagulant treatment (warfarin), as per the recent ESC guidelines,^4^ has proven effective, with reduced thrombus size observed in CMR imaging follow-up.

## Conclusion

In conclusion, we present a case of incidentally detected late LV mural thrombus after post-extensive anterior STEMI with a background of CML. This case illustrates the superior diagnostic sensitivity of CMR for detecting intracardiac thrombi and highlights the crucial role of CMR in monitoring treatment response and thrombus resolution, particularly in patients with high-risk thrombus formation and suboptimal echocardiographic results.

## Lead author biography


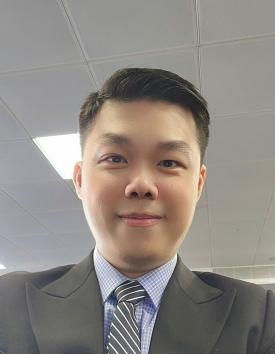


Dr. med. Qui Minh Nguyen is a young internal medicine resident at the Pham Ngoc Thach University and a cardiovascular magnetic resonance fellow at the Royal Brompton Hospital. His primary academic and clinical interests focus on ischaemic heart disease and cardiomyopathy (e.g., amyloidosis, dilated cardiomyopathy), aiming to contribute to advancements in the understanding and treating cardiomyopathy and ischaemic heart disease.

## Supplementary Material

ytaf615_Supplementary_Data

## Data Availability

The data used in this case are available in the article and online Supplementary material.

## Notes

**Consent:** The authors confirm that written consent for submission and publication of this case report, including images and associated text, has been obtained from the patient in line with COPE guidance.

**Funding:** None declared.
